# Population Genetic Structure of a Microalgal Species under Expansion

**DOI:** 10.1371/journal.pone.0082510

**Published:** 2013-12-12

**Authors:** Karen Lebret, Emma S. Kritzberg, Karin Rengefors

**Affiliations:** Aquatic Ecology, Department of Biology, Lund University, Lund, Sweden; George Washington University, United States of America

## Abstract

Biological invasions often cause major perturbations in the environment and are well studied among macroorganisms. Less is known about invasion by free-living microbes. *Gonyostomum semen* (Raphidophyceae) is a freshwater phytoplankton species that has increased in abundance in Northern Europe since the 1980's and has expanded its habitat range. In this study, we aimed to determine the genetic population structure of *G. semen* in Northern Europe and to what extent it reflects the species' recent expansion. We sampled lakes from 12 locations (11 lakes) in Norway, Sweden and Finland. Multiple strains from each location were genotyped using Amplified Fragment Length Polymorphism (AFLP). We found low differentiation between locations, and low gene diversity within each location. Moreover, there was an absence of genetic isolation with distance (Mantel test, p = 0.50). According to a Bayesian clustering method all the isolates belonged to the same genetic population. Together our data suggest the presence of one metapopulation and an overall low diversity, which is coherent with a recent expansion of *G. semen.*

## Introduction

Invasive species can pose a significant threat to ecosystems with a wide range of effects on the newly colonized environment such as loss of biodiversity, disappearance of native species, shift in species dominance in the community, and alteration of ecosystem function [1]. While biological invasions in terrestrial and aquatic systems have been widely studied for a large range of macroorganisms, few studies focus on invasions of free-living aquatic microbes [2]. The lack of studies on microbial invasions may be partly explained by the fact that microbial invasion are difficult to detect in the environment [2], as the individuals are small and initially low in abundance. Furthermore, microbes were for a long time considered to be cosmopolitan due to their high dispersal capacity and large population sizes [3], and thus not viewed as potential invaders. Although some species are cosmopolitan, it is now known that many have a more restricted geographical distribution [4].

In aquatic systems, microbial planktonic algae (phytoplankton) are key players as primary producers that form the base of the food web in aquatic systems. Studies concerning invasive phytoplankton are scarce, and only a handful of microalgal species are currently described as invasive. These include the marine dinoflagellate *Alexandrium tamarense* [5], the freshwater cyanobacteria *Aphanizomenon ovalisporum* and *Cylindrospermopsis raciborskii* [6], [7], and the freshwater raphidophyte *Gonyostomum semen* [8], [9].

The present study focuses on the raphidophyte species *G. semen*, which is considered a nuisance species and invasive. This species can form dense blooms over extended periods of time reaching cell abundances of 1.5 millions cells per liter, despite very low growth rates [10]. Moreover, *G. semen* expels slimy threads upon mechanical stress which can cause skin irritation on bathers [11], [12]. The abundance and occurrence of this species has increased in Northern Europe during the last four decades [9], [12], [13], and more recently in Poland [14]. In a recent study based on data from the Swedish National Lake Monitoring program, Rengefors *et al.* [9] showed that blooms of *G. semen* have appeared in new lakes during the past twenty years. These findings are in line with the results of a previous study in Finland [13], also showing an expansion of *G. semen* into new lakes. Thus, the species is now considered invasive in Northern Europe. It has been proposed that an invasive species colonizes new habitats, spread quickly, and forms dominant populations [15].

With the expansion of *G. semen* in Northern European lakes, studies exploring the environmental conditions of *G. semen* lakes and describing the species ecological characteristics have been undertaken [8], [12], [16]. For instance, *G. semen* growth has been shown to be favored by humic substances [16]. Hence, the species blooms primarily in brown-water lakes with high concentrations of dissolved organic carbon [12], although it has also be observed in clear-water lakes [17]. In addition, the formation of dense population by *G. semen* has been suggested to be favored by a reduced grazing pressure by zooplankton [8], and its ability to migrate in the water column to acquire nutrients during night and light during day [12]. However, little has been done to explore the population genetic structure of *G. semen* populations, and no study yet attempted to explain the invasion pattern of the species. Presumably, a recent (decades rather than thousands of years) invasion should be reflected in the species population genetic structure. Several studies on phytoplankton population genetics have shown that populations are typically highly differentiated with low gene flow even between neighboring populations [18], [19], [20], [21], [22]. However, most of the studies focused on marine systems and little has been done in limnic systems, which are more isolated, thus more likely to show restricted gene flow. Rengefors *et al*. [21] showed that lakes can act as islands according to the island biogeography theory [23], [24], with the presence of highly differentiated populations on small geographical scale. For invasive phytoplankton, we can expect the populations to show little differentiation due to the short time since separation and low genetic diversity following the bottleneck occurring during colonization and establishment of individuals.

In this study, we aimed at characterizing the population genetic structure and diversity of *G. semen* in Northern Europe to get a better understanding of the species expansion. To this end, we used the DNA fingerprinting method Amplified Fragment Length Polymorphism (AFLP) to determine the population structure and genetic diversity of *G. semen* in 11 lakes spread across Sweden, Finland and Norway.

## Methods

### Study sites and Sampling

**Figure 1:**
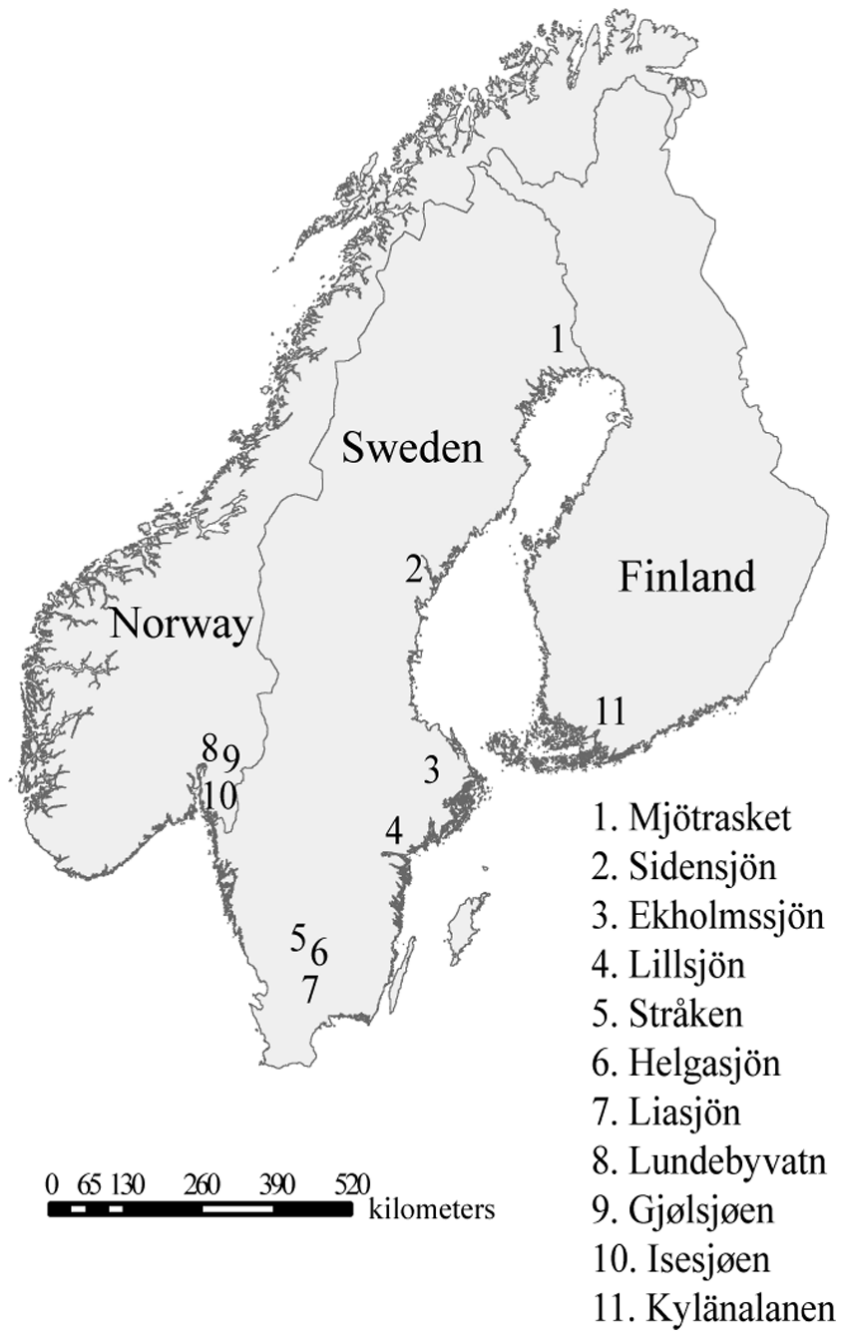
Map representing the sampled lakes.

During summer 2010 (July-August), 12 stations from 11 lakes were sampled across Sweden (7 lakes), Norway (3 lakes), and Finland (1 lake) (Fig. 1, and Table 1). The sampling scheme was designed to cover two major axes: a North-South axis within Sweden; and a West-East axis with samples from Norway, Sweden and Finland. The locations were chosen to obtain a nested design with a wide range of geographical distances, from a few km to more than a thousand km between lakes (Table 2). The largest lake, Helgasjön was sampled at two different sites 10 km apart to determine if different populations co-exist in larger lakes. The selection of the lakes was done using databases from monitoring programs (SYKE for Finland, NIVA for Norway and SLU for Sweden) or from published data. The lakes were known to have recurrent *G. semen* blooms. At each sampling station *G. semen* cells were collected from the shore in the surface water using a 20-µm mesh-size plankton net. Large zooplankton which might feed on *G. semen*, were removed from the sample using a 150-µm net. Water from each lake was collected for preparation of culture medium for the *G. semen* isolates.

**Table 1:** Summary of lake data for each samples lakes (-: data not known).

Lakes	Country	Sampling date	Coordinates	Area (km^2^)	*G. semen* abundance (cells L^−1^)	Monitoring period	Known *G. semen* occurrence years
Mjöträsket	Sweden	6 July	65°55′55″N 23°05′50″E	2.56	4200	2006	2006
Sidensjön		8 July	63°53′12″N 19°48′53″E	0.089	117200	2004–2010	2004–2010
Ekholmssjön		11 Aug	59°52′19″N 17°03′36″E	0.057	34700	1995–2010	1995–2010
Lillsjön		10 Aug	58°48′06″N 17°26′37″E	-	30000	-	-
Stråken		5 Aug	57°07′11″N 14°34′10″E	7.7	26000	-	1948
Helgasjön Station 2		4 Aug	56°58′42″N 14°42′21″E	50.2	3200	-	-
Helgasjön Station 1		4 Aug	56°55′26″N 14°49′24″E	50.2	1800	-	1948
Liasjön		23 July	56°26′78″N 13°59′56″E	0.12	408000	-	-
Lundebyvatn	Norway	28 July	59°32′48″N 11°28′57″E	0.42	286300	2005–2010	2005–2010
Gjølsjøen		28 July	59°26′24″N 11°41′01″E	0.98	14800	-	-
Isesjøen		28 July	59°17′42″N 11°14′40″E	6.35	60200	2005–2010	2005–2010
Kylänalanen	Finland	20 July	60°24′21″N 23°45′01″E	-	5100	-	2004

**Table 2:** Geographic distances in km between sampling locations.

	Mjöträsket	Sidensjön	Ekholmssjön	Lillsjön	Stråken	Helgasjön Stat. 2	Helgasjön Stat. 1	Liasjön	Lundebyvatn	Gjølsjøen	Isesjøen	Kylänalanen
Mjöträsket		371	841	949	1177	1189	1193	1259	1004	1006	1035	727
Sidensjön			470	580	806	818	822	888	652	651	682	438
Ekholmssjön				121	339	350	353	422	315	300	334	375
Lillsjön					253	260	260	333	350	331	359	397
Stråken						18	27	83	325	305	311	643
Helgasjön Stat. 2							9	74	343	323	328	647
Helgasjön Stat. 1								74	351	332	337	645
Liasjön									375	358	356	718
Lundebyvatn										21	31	690
Gjølsjøen											35	675
Isesjøen												709

### Ethics statement

No specific ethical permits were required for the specific study according to the Swedish, Finnish, and Norwegian laws. The locations are not privately-owned or protected in any way.

### Isolation, culture and harvesting of G. semen cells

Single cell isolations were performed to obtain a final number of approximately 20 clonal cultures per sampling location (Table 3). The isolations and culture of the strains were performed according to Lebret *et al.* [10]. The strains of each lake were cultivated in a culture medium, composed of 50% of modified Wright's cryptophyte medium (MWC) [25] with an addition of selenium to a final concentration of 2.5 µg L^−1^ and 50% sterile-filtered water from the respective lakes to increase the isolation success. Survival rates of the strains for each population (in%) were calculated after two months of culture, by dividing the number of strains alive by the number of cells isolated. When the cell concentration of the cultures had reached approximately 2000 cells mL^−1^, the cells were harvested by centrifugation according to Lebret *et al.* [10] and the pellets were frozen at −80°C until DNA extraction.

**Table 3:** Number of strains genotyped, survival rate, Nei's gene diversity within locations and percentage of polymorphic loci for each location.

	Sampling stations	Number of genotyped strains	Survival rates (%)	Nei's gene diversity	Percentage of polymorphic loci
1	Mjöträsket	22	43	0.043	22.1
2	Sidensjön	16	29	0.050	21.0
3	Ekholmssjön	17	51	0.029	12.4
4	Lillsjön	15	35	0.022	8.9
5	Stråken	18	45	0.054	25.4
6a	Helgasjön Stat. 1	8	22	0.049	12.9
6b	Helgasjön Stat. 2	19	50	0.041	19.2
7	Liasjön	13	25	0.023	7.8
8	Lundebyvatn	18	41	0.038	17.9
9	Gjølsjøen	17	36	0.077	32.6
10	Isesjøen	17	42	0.040	16.9
11	Kylänalanen	14	41	0.040	16.0

### DNA extraction

DNA was extracted using a CTAB-based protocol described by Lebret *et al.* [10]. The DNA concentration of the samples was estimated by measuring the absorbance of a subsample diluted ten times at 260 nm using a spectrophotometer (Ultraspec 3000, Pharmacia biotech). For each sample, the quality of the DNA was determined using the 260/280 ratio. Only samples of high DNA quality, i.e. with a 260/280 ratio of 2.0, were used for downstream analyses. The DNA samples were stored at −80°C until genotyping.

### Genotyping by AFLP analysis

Amplified fragment length polymorphism (AFLP) analyses were performed on the samples as described by Lebret *et al.* [10]. For the selective amplification, the M and E-primers were 5′-GACTGCGTACCAATTCNNN-3′, and 5′-GATGAGTCCTGAGTAANNN-3′ respectively. Specifically, the following six primer combinations were used: E_TCT_ x M_CGA_, E_TCT_ x M_CCG_, E_TAG_ x M_CGG_, E_TCG_ x M_CAG_, E_TCG_ x M_CGG_ and E_TCG_ x M_CGA_. PCR products from three primer combinations labeled with the different dyes (Ned, Fam and Hex) were combined in single wells of a 96-well plate (Applied Biosystems). All samples were analyzed by ABI37730XL capillary electrophoresis using a MapMarker 1000 bp size standard at the Uppsala Genome Center, Sweden.

### AFLP data analyses

The raw data was analyzed with Genemapper (Version 4.0, Applied Biosystems) and AFLPscore version 1.4 [26] was used to score the data. Fragments between 50 to 1000 bp were sized and scored. The error rate between replicates was minimized to less than 2.5% for each primer combination based on duplicates of 20 randomly chosen strains according to Whitlock *et al.* [26]. After the scoring, a data set based on presence/absence of fragments was generated using AFLPscore. The data were checked manually to identify identical clones (genotypic diversity). The Nei's gene diversity [27] and the percentage of polymorphic loci were determined for each sampling date using the R script AFLPdat [28]. The data file was converted into input files compatible for Arlequin and STRUCTURE using AFLPdat [28]. Arlequin version 3.5.1.2 [29] was used to calculate pair-wise F_ST_ values to estimate genetic differentiation between sampling locations, the p-values were determined using 1000 permutations, and the F_ST_ were considered significant for p≤0.05. Genetic differentiation was also estimated with Jost's D distance [30] between each location using the program Spade [31]. Confidence intervals of the Jost's D values were calculated with a bootstrap of 1000. The geographic distances between lakes were calculated using the GPS coordinates using the R script AFLPdat [28]. A Mantel test was performed using Arlequin with 1000 iterations to determine the presence of isolation by distance. An analysis of the molecular variance (AMOVA) was performed using Arlequin [29]. Arlequin was also used to determine the presence of loci under selection in the data set, a hierarchical island model was used with 10,000 iterations, loci with p≤0.01 were defined as outliers, meaning potential loci under selection. A principal component analysis based on genetic distances at the population level was performed using the package GENALEX 6.41 in Excel [32] using the binary model for diploid organisms based on the method of Huff *et al*. [33]. The data were not checked for multivariate normality. The number of genetic populations were determined using the software STRUCTURE 2.3.3 [34] without prior information on the sampling location. All the combinations of model settings, admixture or no-admixture ancestry models with either correlated or independent allele frequency, were tested (four models in total). Each run had a burn-in of 50,000 iterations followed by 50,000 iterations of data collections. We tested up to 16 populations (K) with 10 iterations at each level. The results were analyzed according to Evanno *et al.* [35] to identify the number of populations that best fits our dataset.

## Results

A total of 194 strains from 12 sampling stations (11 lakes) across Sweden, Norway and Finland (Fig. 1 and Table 3), were successfully isolated, cultivated, and genotyped (Table 3). The survival rates of the isolates ranged from 22 to 51%, (Table 3). 614 AFLP loci were retained for the population genetic analyses. No identical genotypes were observed in the entire dataset, i.e. all the AFLP profiles were different. Nei's gene diversity ranged from 0.022 (Lillsjön) to 0.077 (Gjølsjøen; Table 3). The percentage of polymorphic loci within location varied between 7.8% (Liasjön) and 32.6% (Gjølsjøen; Table 3).

**Figure 2:**
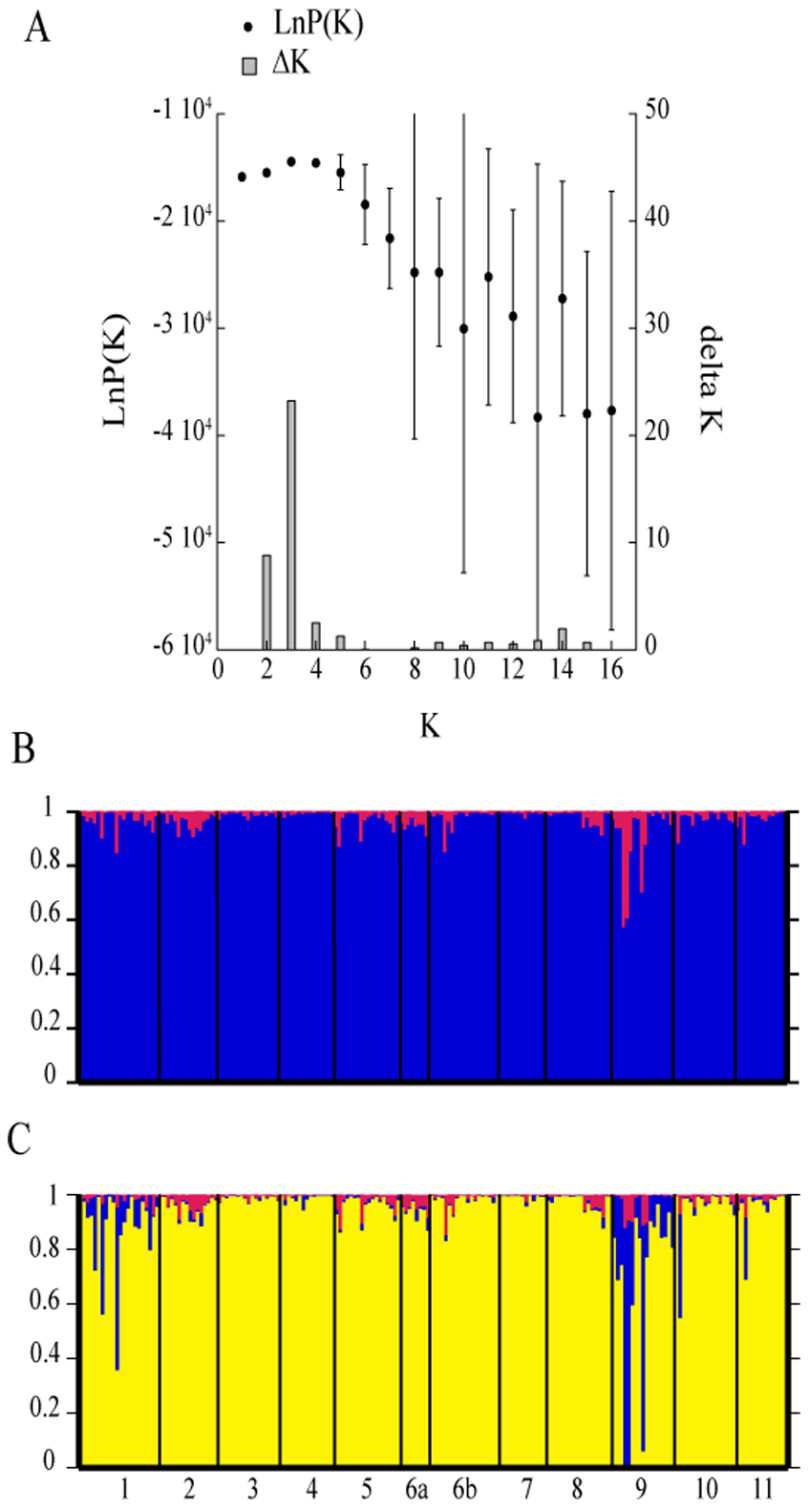
Summary of the results from the STRUCTURE analysis. The analysis was performed using the admixture model allowing for correlated frequencies: Graph of LnP(K) (black dots) and ΔK (bars) for the different K population assumptions (A). Bar plots representing the population assignment of the individuals for the assumption of K = 2 (B), K = 3 (C).

To analyze the STRUCTURE results, the number of populations (K) was determined using two approaches. Based on the calculated lnP(K) value (Fig. 2A), the smallest K on the plateau is the correct number, i.e. K = 1 in this case. According to the method recommended by Evanno *et al.* [35], two or three populations could be identified because of the presence of a peak of ΔK (23.3) at K = 3, and a high ΔK (8.8) at K equal 2 (Fig. 2A). For the simulations of K = 2 and K = 3, the strains were assigned to the same population (>95% of assignment, Fig. 2B and C). Only Gjølsjøen had strains that were assigned to more than one population, suggesting a more diverse population within this lake. Thus, the STRUCTURE results best support the presence of one dominant genetic population across all our sampling points. Analysis of molecular variance (AMOVA) showed that 81% of the variation was observed within locations, and only 19% by variation among locations.

All the pairwise F_ST_ between sampling locations were statistically highly significant, although low. The Mantel test showed that the differentiation between the lakes could not be explained by genetic isolation due to geographic distances (P = 0.50). The highest F_ST_ was observed between the samples collected in Helgasjön 1 and Lillsjön (F_ST_ = 0.306; Table 4). The lowest value was observed between Helgasjön 2 and Stråken with F_ST_ equal to 0.048 (Table 4). In general, Stråken showed the lowest F_ST_ values, and Helgasjön station 1 the highest ones, although the sample size of Helgasjön station 1 was low, thus this result should be interpreted with caution. The genetic differentiation between sampling locations was also estimated using Jost's D distances. All Jost's D distances were equal to zero or low between the locations (Table 4). The highest differentiation was observed between Gjølsjøen and Lillsjön (D = 0.058). The confidence intervals include zero for all the pairwise comparisons at the exception of the D values between Gjølsjøen-Lillsjön and Gjølsjøen-Mjöträsket, suggesting an absence of genetic differentiation between most of the sampling locations.

**Table 4:** Genetic differentiation expressed as pair-wised F_ST_ (below the diagonal; ** denotes p<0.01, *** denotes p<0.001), and Jost's D (above the diagonal; with confidence intervals between brackets) between sampling locations.

	Mjöträsket	Sidensjön	Ekholmssjön	Lillsjön	Stråken	Helgasjön Stat. 2	Helgasjön Stat. 1	Liasjön	Lundebyvatn	Gjølsjøen	Isesjøen	Kylänalanen
Mjöträsket		0.010 (0–0.041)	0.008 (0–0.039)	0 (0–0.029)	0 (0–0.021)	0 (0–0.017)	0.007 (0–0.061)	0 (0–0.019)	0.003 (0–0.034)	0.039 (0.005–0.074)	0.012 (0–0.045)	0 (0–0.012)
Sidensjön	0.155^***^		0 (0–0.022)	0 (0–0.030)	0 (0–0)	0 (0–0.002)	0 (0–0.032)	0 (0–0.029)	0 (0–0.013)	0.002 (0–0.037)	0 (0–0.028)	0 (0–0.004)
Ekholmssjön	0.182^***^	0.136^***^		0 (0–0.009)	0 (0–0.018)	0 (0–0)	0.001 (0–0.052)	0 (0–0.010)	0 (0–0.024)	0.030 (0–0.068)	0 (0–0.020)	0 (0–0.010)
Lillsjön	0.167^***^	0.165^***^	0.181^***^		0.001 (0–0.034)	0 (0–0.012)	0.006 (0–0.057)	0 (0–0.003)	0.006 (0–0.043)	0.058 (0.022–0.099)	0.012 (0–0.048)	0 (0–0.003)
Stråken	0.101^***^	0.068^***^	0.114^***^	0.150^***^		0 (0–0)	0 (0–0.022)	0 (0–0.019)	0 (0–0.004)	0 (0–0.026)	0 (0–0.025)	0 (0–0)
Helgasjön Stat. 2	0.109^***^	0.082^***^	0.095^***^	0.150^***^	0.048^***^		0 (0–0.012)	0 (0–0.003)	0 (0–0)	0 (0–0.035)	0 (0–0.013)	0 (0–0)
Helgasjön Stat. 1	0.220^***^	0.166^***^	0.271^***^	0.306^***^	0.134^***^	0.140^***^		0.005 (0–0.061)	0 (0–0.053)	0 (0–0.046)	0.006 (0–0.065)	0 (0–0.031)
Liasjön	0.139^***^	0.165^***^	0.185^***^	0.205^***^	0.118^***^	0.124^***^	0.301^***^		0 (0–0.013)	0.030 (0–0.074)	0 (0–0.021)	0 (0–0)
Lundebyvatn	0.153^***^	0.111^***^	0.178^***^	0.233^***^	0.075^***^	0.093^***^	0.237^***^	0.159^***^		0.006 (0–0.041)	0 (0–0.019)	0 (0–0.004)
Gjølsjøen	0.157^***^	0.112^***^	0.169^***^	0.209^***^	0.087^***^	0.104^***^	0.132^**^	0.162^***^	0.124^***^		0.011 (0–0.045)	0 (0–0.034)
Isesjøen	0.167^***^	0.134^***^	0.158^***^	0.241^***^	0.114^***^	0.113^***^	0.234^***^	0.172^***^	0.139^***^	0.116^***^		0 (0–0.015)
Kylänalanen	0.115^***^	0.103^***^	0.150^***^	0.144^***^	0.071^***^	0.064^***^	0.192^***^	0.132^***^	0.115^***^	0.110^***^	0.142^***^	

**Figure 3:**
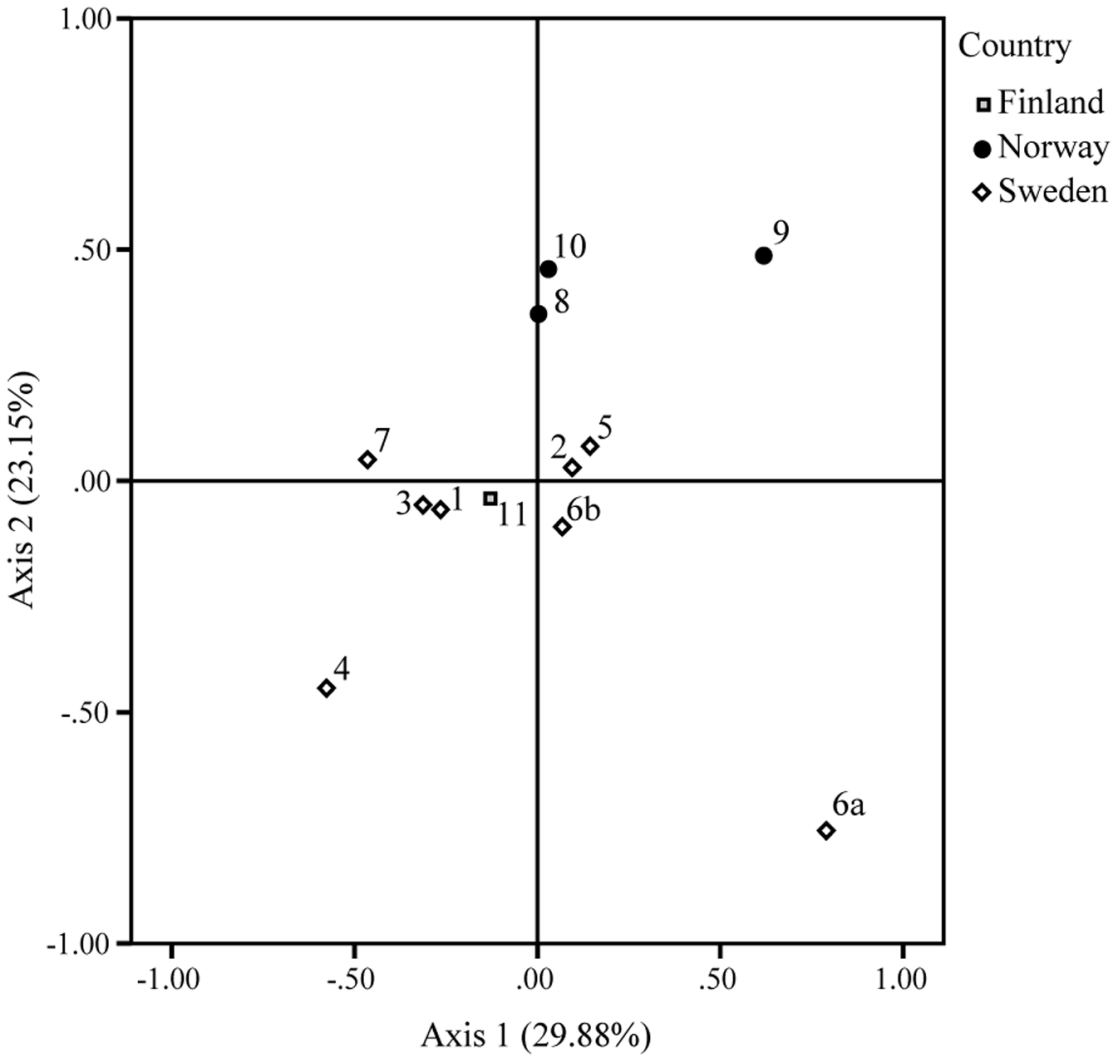
Principal component analysis (PCA) of the genetic distances of the sampled locations. The axes 1 and 2 explained 53% of the variance in distribution of the lake populations (1: Mjöträsket; 2: Sidensjön; 3: Ekholmssjön; 4: Lillsjön; 5: Stråken; 6a: Helgasjön Stat. 1; 6b: Helgasjön Stat. 2; 7: Liasjön; 8: Lundebyvatn; 9: Gjølsjøen; 10: Isesjøen; 11: Kylänalanen).

The results of the principal component analysis (PCA) based on genetic distances, showed that the first axis explained 30% of the observed variation, the second 23% and the third 15% (Fig. 3). Although, the efficiency of the PCA was low, a pattern can be observed. Hence, along the 1^st^ axis, Lillsjön, Gjølsjøen and Helgasjön 1 clustered separately from all the other lakes. The Norwegian lakes were separated from the other lakes along the 2^nd^ axis. These results suggest the presence of a weak east-west geographic pattern of differentiation. However, the PCA was not highly supported, as the first two axes explaining only 53% of the observed pattern. Moreover, Lillsjön and Helgasjön station 1 were genetically separated from the other lakes. Helgasjön station 1 was the most genetically distant lake, in accordance with the differentiation observed with the F_ST_ values. The other Swedish and Finnish lakes grouped together, indicating that the lakes were not genetically distant from each other (Fig. 3). Along the 3^rd^ axis, all the lakes grouped together except for Mjöträsket in Northern Sweden.

We performed an outlier analysis in Arlequin to determine if our dataset contained loci that might be under selection. The outlier analysis showed that 8 loci were potentially under selection pressure. Four of these loci were only present in the strains collected from Helgasjön station 1. Single unique loci were only observed in the Mjöträsket, Lillsjön, Isesjøen, and Gjølsjøen strains respectively.

## Discussion

Previous studies have revealed that *G. semen* shows signs of range expansion in Northern Europe, and the species has been described as invasive [9], [13]. In this study we have used a population genetic approach to characterize the population structure of *G. semen*, to gain a better understanding of patterns of invasion. Our results showed the presence of a single metapopulation (STRUCTURE and AMOVA analyses) in Northern Europe, with low differentiation between sampling locations. The results are discussed below in light of the recent expansion (during the last decades) of the species in Northern Europe.

According to population genetic theory, a recently established population is expected to have low diversity because most likely only a fraction of the source population would have dispersed, generating a diversity bottleneck in the new population [36]. In addition, invasive species are expected to show weak or low population structure [37]. This is because dispersed individuals that have established and formed the invasive populations likely originate from the same population or from closely related populations. Previous studies on invasive species showed that genetic variation was mostly explained by within population variation in the invaded areas, showing a lack of population structure in the invasive range [38], [39]. The results from our study on *G. semen* are consistent with these patterns.

The presence of one single metapopulation (STRUCTURE and AMOVA analyses) over large distances, most likely reflect the recent invasion, as more differentiated populations could be expected from non-invasive phytoplankton populations. For instance, F_ST_ values between two phytoplankton populations identified by STRUCTURE typically range ≥0.22–0.4 in other studies [21], [40], which is despite some overlap overall slightly higher than found in the present study (F_ST_ ranged between 0.048 and 0.306). Similarly, Logares *et al.* [41] showed the presence of two distinct populations (using STRUCTURE analysis on AFLP data) of the dinoflagellate *Peridinium aciculiferum* in two Swedish lakes.

Although the STRUCTURE analysis suggests the presence of a single population in Northern Europe, there was still a significant but low genetic differentiation (based on F_ST_) among the lakes. This discrepancy can be explained by the different approaches and assumptions of F_ST_ and STRUCTURE to analyze population structure. The STRUCTURE analysis assigns individuals to potential populations assuming that the populations are in Hardy-Weinberg equilibrium, whereas the F_ST_ analyzes the heterozygosity between pre-defined populations. Thus, the STRUCTURE analysis is often considered more conservative, and might detect higher level of population structure. However, the statistical significance observed for our F_ST_ values between locations might result from low within location variation, rather than reflecting strong differentiation. The statistical differentiation observed for the F_ST_ analysis can also result from the presence of a few loci only observed in specific sampling locations (loci under selection analysis). In fact, our data does show the presence of several loci that may be under selection, which are only present in specific lakes. These results need to be interpreted with caution, as they can result from false-positive or hitchhiking phenomenon, where alleles appear to be under selection due to linkage to loci under true selection [42]. Further investigations are needed to understand the role of neutral versus selected loci in phytoplankton populations. To complement the F_ST_ and STRUCTURE analyses, we also determined Jost's D distance as a measure of population differentiation [30]. Previous studies have suggested that Jost's D distance might have less limitation than F_ST_ analyses, thus being more relevant to study population differentiation [30], [43]. We found pairwise Jost's D distances equal to zero or low, indicating an absence of differentiation of the population of *G. semen* between the different locations. The combination of low F_ST_ values and the absence of differentiation according to Jost's D distance suggest that there is little or no differentiation between the sampling locations, thereby supporting the results of the STRUCTURE analysis.

The lack of genetic differentiation among lakes and low genetic diversity within lakes are best explained by a recent colonization of the lakes, which has likely occurred during the last decades or century as suggested by the monitoring data [9], [13]. Nevertheless, it is very challenging with the data at hand to determine with certainty when the colonization has occurred. If *G. semen* had colonized the lakes following the last glaciation period (several thousands to 10,000 years ago), we would have expected to find high differentiation of populations with a pattern of isolation by distance [44], [45]. In this scenario, *G. semen* would have colonized the lakes gradually from South to North following the receding of the ice cap. Our data does not support the latter hypothesis, thus we conclude that our results reflect the recent (past decades) invasion of lakes.

There are, however, alternative explanations for our results without invoking a recent invasion. A single metapopulation could also have resulted by high gene flow among lakes in *G. semen* even if the lakes were colonized thousands of years ago. However, that explanation seems less likely as monitoring data suggest that *G. semen* has colonized new lakes [9], [13] and that we are observing a true expansion rather than an increase in population sizes. Nevertheless, the possibility that *G. semen*'s presence was previously undetected due to very low abundance in cell numbers remains. For example, a recent study showed that the freshwater diatom *Stephanodiscus binderanus*, which was described as an exotic species in the Great lakes since the mid-20^th^ century, was already present in these lakes since a least three centuries [46]. That study shows that a species might be native although other evidence suggested that it was introduced recently. In our study, while the results of our genetic analyses are coherent with a species under expansion, and support the results from the monitoring studies, a recent increase of abundance of a population that colonized long ago cannot be completely ruled out.

Invasive species are expected to present lower diversity in the invasive range than in the native range [47], [48], [49]. This theory is called the paradox of invasion biology, and suggests that invasive populations should have lower genetic diversity, and consequently have lower capacity to adapt to new conditions than source populations. *G. semen* showed low gene diversity compared to other phytoplankton population studies. For example, freshwater dinoflagellates had a Nei's gene diversity between 0.07 and 0.37 [41], compared to 0.02–0.08 in this study. However, to date, there are few studies on population genetics in phytoplankton species and the level of diversity that can be expected in phytoplankton populations is still unclear. In addition, phytoplankton typically reproduce asexually during their active stage, but sexual events also occur regularly. While asexual reproduction will not alter the genetic diversity, sexual events are important in creating diversity through recombination and by allowing the spreading of favorable alleles [50]. *G. semen* alternates between asexual and sexual reproduction, with a mainly asexual phase during the growing season followed by sexual reproduction at the termination of the bloom [51]. In *G. semen*, the low gene diversity observed in the different populations might be explained by a bottleneck effect resulting from colonization by few individuals, which was maintained in the populations due to the life cycle characteristics involving vegetative growth. However, the gene diversity data alone cannot rule out that the observed diversity is not due to an increase in population sizes of populations that were already present. Nor do we have data from a native range to compare with that of the current range.

In our data set, the lakes Stråken and Helgasjön (Station 1) are the lakes with the oldest known *G. semen* blooms (first reported in 1948, by Sörensen [11]). However, the genetic diversity of these lakes is not very different from that in the other lakes although they are towards the higher end. This phenomenon cannot be explained with the current data set, since phytoplankton data is lacking from this time period (1940's) in the other lakes.

A further complication is that the measured diversities are most likely underestimates of the real diversity as it was calculated using the strains that were able to grow in culture. Isolation and culture techniques are known to be a form of selection of strains [52]. In order to minimize the selection effect and increase our isolation success we used a mix of artificial culture medium and sterile lake water from the respective lakes. Yet, the culture bias cannot be circumvented, as culture of isolates is currently necessary to perform population genetic studies of microorganisms.

In conclusion, our results showed the presence of a single metapopulation in Northern Europe with low differentiation of *G. semen* populations from the different locations, and relatively low gene diversity. We suggest that the genetic pattern observed in this study might reflect the recent expansion of *G. semen* (during the last decades), and the colonization of new lakes.
